# [RETRACTED ARTICLE]: Distribution and association between environmental and clinical isolates of *Cryptococcus neoformans* in Bogotá-Colombia, 2012-2015

**DOI:** 10.1590/0074-02760160560

**Published:** 2017-11

**Distribution and association between environmental and clinical isolates of Cryptococcus neoformans in Bogotá-Colombia, 2012-2015**

**Norida Vélez, Patricia Escandón/+**

Grupo de Microbiología, Instituto Nacional de Salud, Bogotá, Colombia

Following authors’ decision, the article “Distribution and association between environmental and clinical isolates of Cryptococcus neoformans in Bogotá-Colombia, 2012-2015” published in Memorias do Instituto do Oswaldo Cruz 111(10): 642-648, 2016, DOI: 10.1590/0074-02760160201, authored by N Vélez & P Escandón was retracted due to an authorship conflict.

